# Main vocal complaints of elderly patients after leprosy treatment

**DOI:** 10.1590/S1808-86942010000200003

**Published:** 2015-10-19

**Authors:** Francisco Xavier Palheta Neto, Manoel da Silva Filho, José Mariano Soriano Pantoja, Larissa Lane Cardoso Teixeira, Rafaela Vale de Miranda, Angélica Cristina Pezzin Palheta

**Affiliations:** MSc in Otolaryngology - Federal University of Rio de janeiro - UFRJ and PhD in Neurosciences - Federal University of -UFPA., Assistant Professor - Federal University of Pará - UFPA and Pará State University -UEPA. Preceptor at the Medical Residency in Otolaryngology - Betina Ferro de Souza University Hospital; Associate Professor of Biophysics -UFPA; 4th Year Medical Student - UEPA; 4th Year Medical Student - UEPA; 4th Year Medical Student - UEPA; MSc in Otolaryngology - UFRJ; PhD in Neurosciences - UFPA. Assistant Professor - UEPA. Preceptor at the Medical Residency Program in Otolaryngology - Betina Ferro de Souza University Hospital

**Keywords:** voice disorders, leprosy, aged, quality of life, hoarseness

## Abstract

Leprosy is an infectious disease, with vocal involvement varying between hoarseness and difficult breathing.

**Aim:**

compare the main vocal complaints among elderly patients after treatment for leprosy and a control group.

**Study design:**

descriptive prospective.

**Materials and methods:**

We included 50 patients aged over 60 years, 32 had been treated for leprosy, and the others formed the control group. We used our own questionnaire to analyze the vocal symptoms presented by the two groups, as well as gender, age, life style and comorbidities.

**Results:**

among the treated group, the most frequent symptoms were hawk (34.4%) and hoarseness (28.1%), while in the control group the most prevalent symptoms were hoarseness (77.8%) and a foreign body sensation (55.6%).

**Conclusion:**

the most prevalent voice complaints in patients treated for leprosy are hawking and hoarseness, and that its development is influenced by life style and associated diseases.

## INTRODUCTION

Speaking requires a precise adaptation of the speech organs so as not to have dysphonic symptoms which could impair the individual's performance in society^1^. Such symptoms can stem from inadequate or excessive use of one's voice, or even from morpho-functional alterations in the larynx-voice set, such as what we have in numerous infectious-contagious diseases, like leprosy. This disease is spread throughout the world and Brazil is among the five most endemic - today we have a prevalence of 4.6/10,000 cases reported in the beginning of 2004.

In relation to the disease's nasal manifestations, the mucosal involvement has a descending characteristic, in other words, it starts in the nasal cavities and, after that, it goes to the oropharynx and larynx6. The initial laryngeal involvement happens in the supraglottis, especially the epiglottis, with erythema and edema evolving and involving the glottis without pain. The characteristic nodules develop and later on ulcerate, forming a scar tissue which can cause stenosis^5^.

Laryngeal involvement is manifested from hoarseness all the way to respiratory distress by edema in the epiglottis, arytenoids and vestibular folds because this is a granulomatous disease. There is also nasal septum perforation, nasal wing collapse and, consequently, nasal deformity. Among nasal manifestations there is hyper secretion with crusts, ulcers and mucosal drying^5^.

In leprosy it is common to see, whether by primary involvement of the larynx and vocal fold, or by a morpho-functional alteration of the important resonating elements involved in the vocal production process.

Usually, in the elderly, there are two major morphology alterations in the laryngeal framework - Calcification and gradual ossification of the laryngeal cartilages. The structure in layers of the vocal folds also suffers alterations in relation to the fibers, causing a reduced mobility and progressive laryngeal muscle atrophy, leading to presbyphonia^8^.

Presbyphonia onset, its development and degree of deterioration depend on each individual, physical and psychological health, and body factors, inheritance, nutritional aspects, social and environmental factors such as smoking and drinking^8^. The main complaints are vocal quality alterations such as hoarseness and aphonia; vocal fatigue; vocal tremor; a feeling of burning or foreign body sensation in the larynx^9^. The vocal alterations caused by leprosy worsen the expected vocal deficit perceived in the elderly, thus impairing interpersonal relations^7^.

Thus, it is important to do a complete otorhinolaryngological exam as a routine in patients with leprosy, especially the elderly, enhancing the accuracy in the identification of the lesions which were not previously seen, in ruling out other alterations with signs and symptoms similar to leprosy such as presbyphonia, and also to enable or aide in the diagnosis in association with the clinical history, and efficiently treat before there are alterations which make the patient stigmatized^4^^,^^6^.

Within such context, the present study aims at outlining the main vocal complaints of elderly patients after being treated for leprosy.

## MATERIALS AND METHODS

All the subjects in the present investigation were studies according to the Helsinki Declaration and the Nuremberg Code, respecting the guidelines for research with human beings (RES. CNS196/96) of the National Health Council, after being approved by the Ethics in Research with Human Beings Committee and proper authorization from the owner of the place where the data was collected, also being duly authorized by the subjects in it by signing the Free and Informed Consent Form. The project was appreciated and approved by the Ethics in Research with Human Beings Committee of the Federal University of Pará (CEP- ICS/UFPA), and the researcher in charge was contacted through e-mail on May 09, 2008.

We included 50 patients, from both genders, with ages above 60 years. From these, 32 had prior history of leprosy and had vocal impairment. The remaining ones made up the control group, with 18 patients with 60 years of age or older, without prior history of leprosy.

In the study group we included the elderly with more than 60 years and prior history of leprosy. All were submitted to videolaryngoscopy and acoustic voice analysis. We took out those who had professions which required constant use of one's voice, the so-called “voice professionals”, such as: teachers, telemarketing operators, radio hosts, singers and religious pastors, amongst others.

The control group was randomly formed, according to the individual's acceptance to participate in the study, with the requirement of not being a “voice professional” or having any other laryngeal involvement because of benign or malignant diseases.

Data collection was held through the use of our own questionnaire (Attachment I), where there is information such as age, disease duration, treatment duration, main signs and symptoms, comorbidities and life style. We chose not to use already validated questionnaires because none of them punctually approaches such diverse aspects of the patient at hand.

Later on, we set up a Microsoft Excel 2007 data bank. We used the comparative statistical analysis through the chi-squared test. In all the tests we established 0.05 or 5% as the rate to reject the null hypothesis, and the asterisk (*) was used to check the significant values.

**ATTACHMENT - RESEARCH PROTOCOL**

**IDENTIFICATION**

1. Name:___________________________________________________________

2. Assessment date: _______ / _______ / _______ 3. Type of leprosy: ______________

4. Telephone: ___________ 5. Age: _______ years 6. Gender: ( ) M ( ) F

**CURRENT DISEASE HISTORY**

1. Under treatment? ( ) no ( ) yes. For how long? _______ ( ) finished. When? _______

2. Feels throat pain or tenderness? ( ) no ( ) yes

3. Hoarseness? ( ) no ( ) yes

( ) constant ( ) constant with floating ( ) in episodes

4. Feels the need of hawking? ( ) no ( ) yes

5. Foreign body sensation in the throat: ( ) no ( ) yes

6. Feels neck pain? ( ) no ( ) yes

7. Keeps any care or medication for the throat or voice?

( ) No ( ) Yes Which? _________________________________________________

8. HBP? ( ) no ( ) yes 9. DM? ( ) no ( ) yes 10. Cardiopathy? ( ) no ( ) yes

11. GERD? ( ) no ( ) yes

**LIFE QUALITY / HABITS / STYLE**

1. As to water ingestion (hydration), how do you classify yourself:

( ) drinks little (forgets or does not feel thirsty)

( ) drinks moderately (1 to 2 litters per day)

( ) drinks a lot (more than 2 litters per day)

2. Do you smoke? ( ) no ( ) yes. How many/day? _______ For how long? _______

( ) former smoker (more than 6 months without smoking)

3. Do you drink alcoholic beverage? ( ) no ( ) yes ( ) former drinker

## RESULTS

The present study was made up of 32 elderly patients with leprosy, of which 65.6% were males and 34.4% were females.

**Graph 1:**
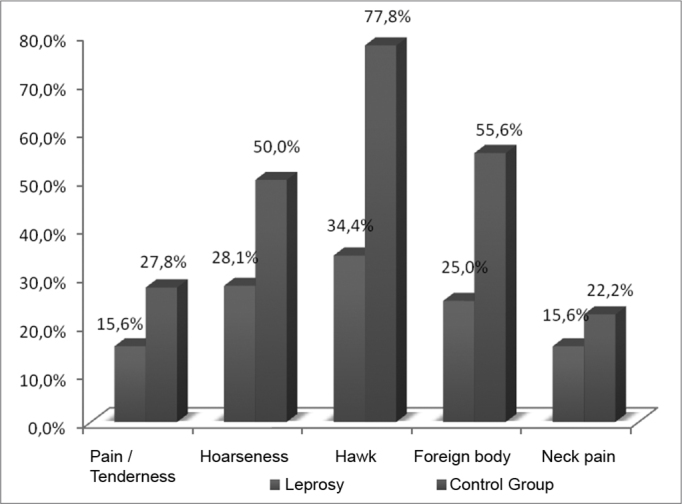
Main vocal symptoms reported by the patients with a past of leprosy and control group, 2008.

Source: Field study.

The main vocal symptoms reported by the patients after the leprosy treatment were hawk (34.4%) and hoarseness (28.1%), followed by a foreign body sensation in the throat (25%), pain/tenderness (15.6%) and neck pain (15.6%) (Table 1, Graph 1). In the control group, hawk was also the most prevalent symptom (77.8%), followed by a foreign body sensation (55.6%), hoarseness (50%), pain/tenderness (27.8%) and neck pain (22.2%) (Table 1, Graph 1).

**Table 1:** Main vocal symptoms reported by the patients with a past of leprosy and control group, 2008.

Symptoms	Leprosy	Control Group	p
Pain / Tenderness	15.6%	27.8%	0.4058
Hoarseness	28.1%	50.0%	0.2979
Hawk	34.4%	77.8%	0.0988
Foreign body	25.0%	55.6%	0.1483
Neck pain	15.6%	22.2%	0.6296

**Graph 2:**
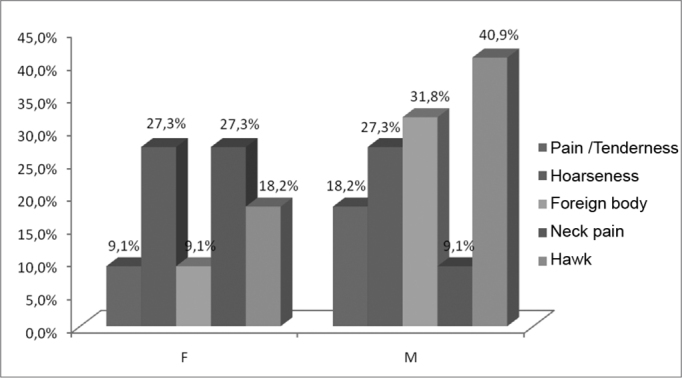
Main vocal symptoms, according to gender, reported by patients with a past of leprosy, 2008.

Source: Field study.

As we analyzed the symptoms associated with gender, among patients treated for leprosy, neck pain and hoarseness proved prevalent among women (both with 27.3%), as well as hawking (40.9 %) and the foreign body sensation (31.8%), were among the most frequent symptoms among men (Table 2, Graph 2).

**Table 2:** Main vocal symptoms, according to gender, reported by the patients with a history of leprosy, 2008.

Gender	Pain / Tenderness	Hoarseness	Foreign body	Neck pain	Hawk
F	9.1%	27.3%	9.1%	27.3%	18.2%
MARISA	18.2%	27.3%	31.8%	9.1%	40.9%

**Graph 3:**
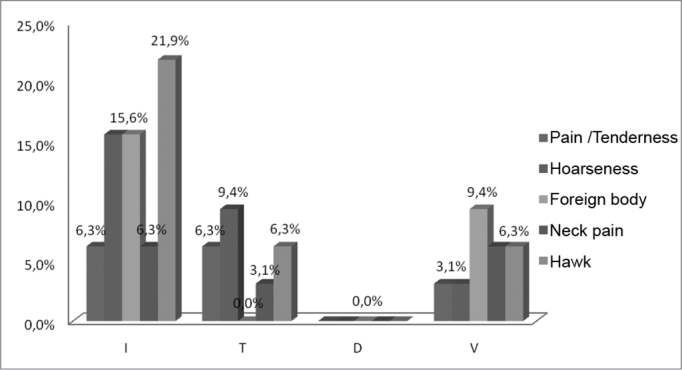
Main vocal symptoms present in each type of leprosy, 2008.

Source: Field study.

The predominant type of leprosy among the patients in this study was the undetermined (56.25%), and the main symptom reported was hawking (21.9%), followed by a foreign body sensation (15.6%) and hoarseness (15.6%). As far as the tuberculoid type is concerned, all the patients mentioned at least one of the symptoms, except for the foreign body. In such type of leprosy, the most frequent symptom was hoarseness (9.4%), followed by hawking (6.3%), pain/tenderness (6.3%) and neck pain (3.1%). Those patients with dimorphous leprosy did not report any of the symptoms studied, while in individuals with the lepromatous form, all the symptoms were present, and the foreign body sensation (9.4%) was the most prevalent, followed by neck pain (6.3%), hawking (6.3%), hoarseness (3.1%) and pain/tenderness (3.1%) (Table 3, Graph 3).

**Table 3:** Main vocal symptoms present in each type of leprosy, 2008.

Types	Pain/Tenderness	Hoarseness	Foreign body	Neck pain	Hawk
I	6.3%	15.6%	15.6%	6.3%	21.9%
T	6.3%	9.4%	0.0%	3.1%	6.3%
D	0.0%	0.0%	0.0%	0.0%	0.0%
V	3.1%	3.1%	9.4%	6.3%	6.3%

**Graph 4:**
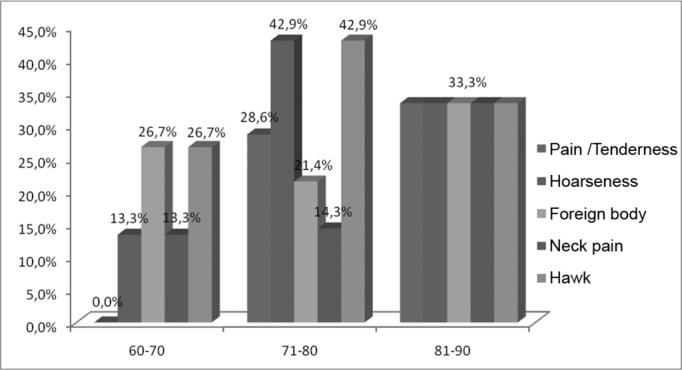
Main vocal symptoms distributed by age range, reported by patients with a past of leprosy, 2008.

Source: Field study.

**Graph 5:**
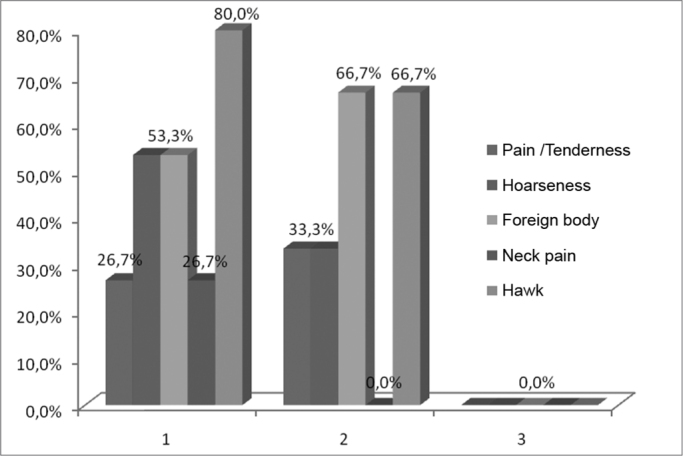
Main vocal symptoms, distributed by age, reported by patients from the control group, 2008.

Source: Field study.

As to age, most patients post leprosy treatment were between 60–70 years (46.8%), followed by 43.7% between 71–80 years, and only 9.5% in the range between 81–90 years. As far as the symptoms presented by age range is concerned, both the patients between 60–70 years and those between 71–80 years, mentioned hawking, a foreign body sensation and hoarseness as the most prevalent symptoms (Table 4, Graph 4). Analyzing the control group, we observed that among the patients between 60–70 years and those between 71–80 years, the most frequent vocal complaints were also hawking, foreign body sensation and hoarseness (Table 5, Graph 5).

**Table 4:** Main vocal symptoms, distributed by age, reported by the patients with a past of leprosy, 2008.

Age range	Pain/tenderness	Hoarseness	Foreign body	Neck pain	Hawk
60–70	0.0%	13.3%	26.7%	13.3%	26.7%
71–80	28.6%	42.9%	21.4%	14.3%	42.9%
81–90	33.3%	33.3%	33.3%	33.3%	33.3%

**Table 5:** Main vocal symptoms, distributed by age, reported by the patients in the control group, 2008.

Age range	Pain/tenderness	Hoarseness	Foreign body	Neck pain	Hawk
60–70	26,7%	53,3%	53,3%	26,7%	80,0%
71–80	33,3%	33,3%	66,7%	0,0%	66,7%
81–90	0,0%	0,0%	0,0%	0,0%	0,0%

**Graph 6:**
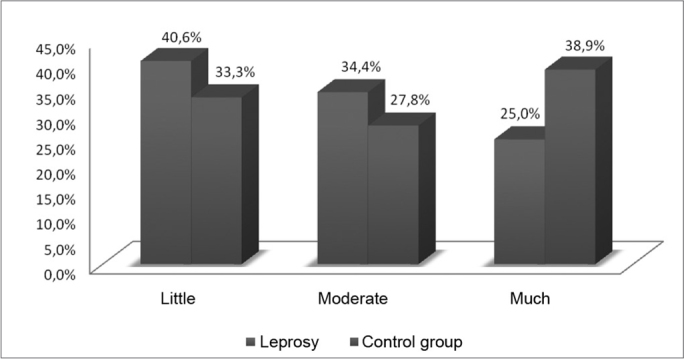
Water intake among the patients with past of leprosy and control group, 2008.

Source: Field study.

In the group of patients post leprosy treatment, most of the patients were former smokers or even with the habit of smoking, 40.6% had never smoked. In this same group, as we observed the symptoms between former smokers and smokers, both mentioning hawk as the most prevalent vocal symptom (Table 6, Graph 6).

**Table 6:** Water consumption among the patients with a past of leprosy and control group, 2008.

Life style	Leprosy	Control group
H20		
Little	40,6%	33,3%
Moderate	34,4%	27,8%
Much	25,0%	38,9%

**Graph 7:**
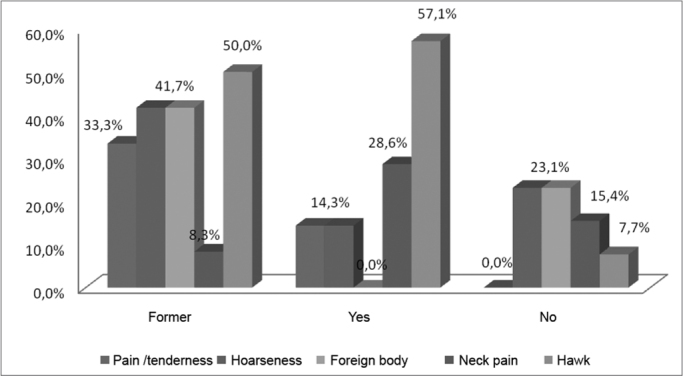
Main vocal symptoms reported by smokers, within the group of patients with a past of leprosy, 2008.

Source: Field study.

Of the elderly patients post-treatment, most of them (40.6%) stated they drank little amounts of water (Table 7, Graph 7). Among the patients in the control group, most of them (38.9%) stated they drank a lot of water (more than two litters per day).

**Table 7:** Main vocal symptoms reported by smokers, within the group of patients with a past of leprosy, 2008.

Smoker	Pain/tenderness	Hoarseness	Foreign body	Neck pain	Hawk
Former	33.3%	41.7%	41.7%	8.3%	50.0%
Yes	14.3%	14.3%	0.0%	28.6%	57.1%
No	0.0%	23.1%	23.1%	15.4%	7.7%

As far as comorbidities are concerned, among the patients after leprosy treatment, 25% had systemic arterial hypertension (SAH), 15.6% had gastroesophageal reflux disease (GERD) and the 12.5% prevalence was equal among heart patients and those with diabetes mellitus (DM). In the control group, the most prevalent comorbidity wad GERD (33.3), followed by SAH (27.8), heart diseases (11.1) and DM (5.6%).

## DISCUSSION

Among the 32 elderly patients with leprosy in our study, most were males. In a study carried out by Silva et al.^6^, similar results were found, having seen that, of the 80 patients with leprosy studied, 31 were females and 49 males. Other studies also observed this leprosy prevalence difference between the two genders, which is probably associated to the predisposition of men having more interpersonal relations and, therefore, a greater exposure to the disease^10^^,^^11^. Nonetheless, Barbosa^5^ noticed a higher number of females with leprosy, which can be justified by the fact that women go more frequently to the doctor than men.

Silva et al.^6^ reported that the laryngeal involvement in leprosy happens later on, nonetheless, the lesions are severe, usually translating into dysphonia, hoarseness and, especially, pain. Notwithstanding, the findings of the present paper showed hawking and hoarseness as main symptoms, and pain/tenderness was the least reported symptom (Table 1, Graph 1). Soubhia^12^ also observed that one of the main laryngeal-related alterations was hoarseness, besides the fact that leprosy stimulates a greater production of mucous, thus causing hawking during speech, and such result is in agreement with the current paper.

Neck pain and hoarseness were the most prevalent symptoms among women, as well as hawking and the foreign body sensation, which are among the most frequent symptoms in men (Table 2, Graph 2). According to Barbosa^5^, the main alterations in the oral-pharynx-larynx region associated with leprosy were globus pharyngeal (7.5%) and neck pain (5%). Besides the difference in clinical manifestations observed between the two genders, it is possible to detect different experiences in living with leprosy, in the social and individual context between men and women. According to Soubhia^12^, women prefer to abandon their jobs, even before being identified as diseased, while men usually choose to hide their disease, for fear of being fired or forced to retire early. Thus, leprosy causes physical disability and can worsen inequalities.

The leprosy prevailing among the patients in this study was the undetermined form, and the main symptom reported was hawking, followed by a sensation of foreign body in the throat and hoarseness. As to the tuberculoid type, all the patients reported at least one of the symptoms, except for the foreign body sensation (Table 3, Graph 3).

According to Fokkens^13^, the hansenotic invasion rarely appears in the undetermined and tuberculoid types, in disagreement with the present study, since the prevalence of symptoms among the patients with the undetermined type was greater than all the others and patients with the tuberculoid type had a prevalence of symptoms similar to that of other forms.

Patients with dimorphous leprosy did not report any of the symptoms reported, while in individuals with the lepromatous form all the symptoms were present (Table 3, Graph 3). In a study carried out by Silva et al.^6^, the lepromatous type was the one presenting the most clinical signs and symptoms, being responsible for 64% of the complaints, following the dimorphous type, which also presented a significant number of complaints, representing 30% of them. These two observations he made are different from the data obtained, having seen that patients with dimorphous leprosy did not have complaints and the lepromatous had less symptom prevalence than the patients with the undetermined type and in equal proportion to the patients with the tuberculoid type.

As to age, most of the patients after treatment for leprosy were between 60–70 years. These data coincide with the study by Barbosa^5^, which indicates that leprosy affects people of all ages, without restrictions, although it rarely occurs in children. As far as symptoms distributed by age range, patients between 60–70 years and those between 71–80 years mentioned hawking, a foreign body sensation and hoarseness as prevalent symptoms (Table 4, Graph 4). Analyzing the control group we noticed that those patients between 60–70 years and 71–80 years had hawking, foreign body sensation and hoarseness as the most frequent complaints (Table 5, Graph 5).

Thus, we noticed the prevalence of these symptoms both in the group of elderly patients after leprosy treatment, as well as in the control group. According to studies by Silva et al.^6^, smoking and the reduced intake of liquids are some of the main harmful habits which can impair vocal production, as well as worsening pre-existing lesions such as leprosy, for instance. In this context, the study of the life styles of these patients from both groups have fundamental importance.

Within this perspective, we analyzed the life style of the patients' post-leprosy treatment and those in the control group. We noticed that most of the patients after treatment were former smokers or still smoked. In this same group, as we observed the symptoms among smokers and former smokers, both mentioned hawking as the most prevalent vocal symptom (Table 6, Graph 6).

According to Soares et al.^8^, smoking causes a heating up of the vocal folds, which may result in a lower voice, as well as a variety of diseases in the laryngeal structure. Silva et al.^6^ states that smoking is highly irritative, and the smoke acts directly upon the vocal mucosa, causing an intense discharge of mucous, which causes the tissue cilia to stop moving, causing a deposit of secretion which in turn causes hawking. Therefore, smoking can potentialize laryngeal damage caused by leprosy, leading to the vocal complaints observed in this study.

Another factor which can worsen the vocal damage caused by leprosy is the reduced consumption of liquids. Of the elderly patients after treatment, most stated they drank little amounts of water (Table 6, Graph 6). Among the control group patients, most reported they drank a lot of water (more than 2 liters per day). According to Soares et al.^8^, the water ingestion must be emphasized for voice maintenance, and the ideal number is from 4 to 6 glasses of water before intensive use, protecting against vocal fold friction during phonation, and avoiding pathological alterations in the vocal pattern. Vocal symptoms such as hoarseness and especially dryness can be relieved through proper water intake.

Throughout this discussion, we can notice that most of the patients in the control group stated not being a smoker, drinking more than 2 liters of water per day and stated they never drank alcoholic beverages. Therefore, most of the group kept their life styles, mentioned in the literature as prophylactic for vocal symptoms and laryngeal lesions; it can then be concluded that the habits of these patients were not capable of justifying the symptoms they presented. Notwithstanding, besides life style, the literature indicates other factors which can be responsible for vocal symptoms in these patients.

For Soares^8^, aging causes changes to the vocal folds, as well as in other structures associated with voice production. This process of natural voice aging is called presbyphonia. According to Bilton and Sanchez^9^, hoarseness and aphonia, vocal fatigue and the feeling of burning or foreign body in the larynx are among the main complaints and vocal symptoms reported by elderly patients, because of alterations stemming from presbyphonia. Moreover, vocal dysfunctions can also stem from pathological processes, such as inflammatory diseases, neoplasias, laryngeal paralysis and other neurological diseases, as stated by Behlau^14^. Within this perspective, it is possible to pinpoint factors which impact the genesis of hawking, the feeling of foreign body and hoarseness presented by the elderly belonging to the control group.

**Graph 8:**
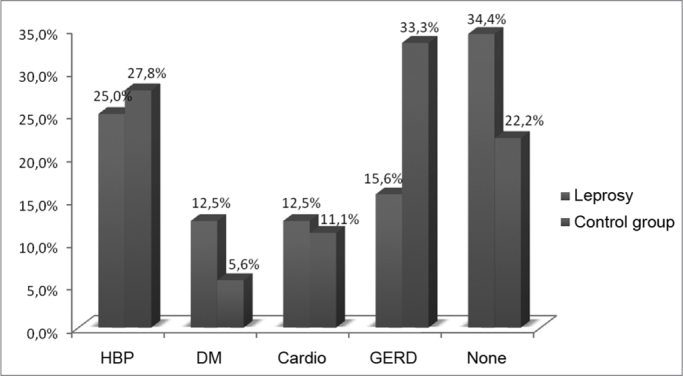
Associated diseases in the patients with a past of leprosy and control group, 2008.

Source: Field study.

The Gastroesophageal Reflux Disorder (GERD) was the most incident morbidity within the control group and, in the group of patients with prior history of leprosy it was the second most frequent co-morbidity (Table 8, Graph 8). This disease is among the main organic causes associated with laryngeal alterations and dysphonia.

**Table 8:** Associated diseases in patients with and without a past of leprosy, 2008.

Associated diseases	Leprosy	Control group
HBP	25.0%	27.8%
DM	12.5%	5.6%
Cardio	12.5%	11.1%
GERD	15.6%	33.3%
None	34.4%	22.2%

Laryngeal damage is likely to occur because of the vagal reflex caused by gastric acid in contact with the esophagus mucosa, triggering reflexes such as clearing the throat and cough which, when chronic, can damage the throat. Another hypothesis to explain the pathogenesis of laryngeal damage in GERD is associated to the direct action of gastrointestinal acid fluids on the larynx because of a malfunction or incoordination of the upper esophageal sphincter.

The classical clinical manifestations more often seen in this disease are: laryngitis, laryngeal stridor, hawking, a foreign body sensation in the throat, dysphagia, hoarseness and chronic cough. Among the control group patients, the most frequent complaints were hawking and a foreign body sensation which can be explained by the considerable percentage of individuals with GERD. Among the patients which were treated for leprosy, there was a greater prevalence of diabetes mellitus when compared to the control group (Table 8, graph 8). According to Behlau^15^, voice production can externalize manifestations of pathological nature, which can be related to metabolism, such as diabetes mellitus. Therefore, we see a relationship between the vocal symptoms presented by both groups and diabetes mellitus, having in mind the importance of the metabolic alterations in the vocal manifestations.

High blood pressure and heart diseases were other comorbidities found in patients with prior history of leprosy (Table 8, Graph 8). Numerous drugs used to treat these diseases can cause vocal alterations. Aspirin, for instance, has anticoagulant properties and, when ingested together with another drug which also contains acetylsalicylic acid or by individuals who have a bleeding disorder, predisposes to a higher risk of vocal fold hemorrhage. Moreover, it causes an increase in blood circulation in the periphery of the vocal folds which, associated to the friction of one vocal fold against the other increases capillary frailty, causing blood spill over to outside the tissues^15^.

In leprosy, the larynx, the epiglottis and the arytenoepiglottic folds are the most frequently affected sites. There is granulomatous edema, which can obstruct the glottis and be translated into aphonia and dyspnea, with a risk of asphixia^4^. These alterations, associated with the vocal changes caused by prolonged use of medication to treat hypertension and/or heart disease, can worsen the damage caused to the patients.

In a nutshell, we noticed that the prevalent symptoms among the patients after treatment for leprosy were hawking and hoarseness, followed by a foreign body sensation, neck pain and tenderness. We also noticed that life styles from most of the patients in this group, very likely collaborated to the appearance and/or worsening of the laryngeal lesions, as well as in the genesis of symptoms presented by them. Among the patients from the control group, life styles proved adequate in relation to what is advocated in the literature. Notwithstanding, the symptoms presented were similar to those found in the group post-leprosy treatment, thus pointing to the existence of other factors which influence the appearance of vocal symptoms.

Based on the analysis developed, we stress the importance of the laryngeal manifestations in leprosy, in order to promote a multiprofessional care to patients with leprosy, where infectologist, dermatologists and otorhinolaryngologists work together to prevent or, at least, to minimize the sequelae of this disease.

## CONCLUSION

We then conclude that the most prevalent vocal symptoms in patients' post-leprosy treatment are hawking and hoarseness, followed by a feeling of foreign body, neck pain and pain/tenderness. We also noticed that most stated they drank little amounts of water besides being smokers or former smokers. The most common comorbidities present were GERD and High Blood Pressure. We could notice that life styles and associated diseases also impact vocal fold symptoms.
